# Spinal arteriovenous fistula coexisting within a spinal lipoma: report of two cases

**DOI:** 10.1038/s41394-017-0011-1

**Published:** 2017-11-14

**Authors:** Yosuke Horiuchi, Akio Iwanami, Takenori Akiyama, Tomohiro Hikata, Kota Watanabe, Mitsuru Yagi, Nobuyuki Fujita, Eijiro Okada, Narihito Nagoshi, Osahiko Tsuji, Ken Ishii, Kazunari Yoshida, Masaya Nakamura, Morio Matsumoto

**Affiliations:** 1 Department of Orthopedic Surgery, Keio University School of Medicine, Tokyo, Japan; 2 Department of Neurosurgery, Keio University School of Medicine, Tokyo, Japan; 3 Department of Orthopedic Surgery, International University of Health and Welfare, Chiba, Japan; 4 Present Address: Department of Orthopedic Surgery, Keio University School of Medicine, 35 Shinanomachi, Shinjuku, Tokyo, 160-8582 Japan

## Abstract

**Introduction:**

Spinal lipoma and spinal arteriovenous fistula (sAVF) are different pathologies and their co-existence is extremely rare. Here we reported two cases of adult-onset sAVF occurring within a spinal lipoma and with review the literature in an attempt to identify the mechanisim of and optimal treatment of this condition.

**Case presentation:**

Case 1 was a 51-year-old man who was treated by embolization of the feeding artery and ligation of the draining vein. Case 2 was a 53-year-old man who was treated by embolization and resection of the tumor containing the shunt zone. In both cases, symptoms improved after surgery. However, in Case 1, angiography at 1 month after the surgery revealed recurrence of the arteriovenous shunt.

**Discussion:**

A literature search revealed only nine other similar case reports. All cases, including ours occurred in adults. In almost all cases, the shunt was located within the spinal lipoma. Pathologic examination revealed venous hypertension, but no evidence of congenital vascular malformation. Given that lipomas release angiogenic factors, the presence of a spinal lipoma may indicate its involvement in the development of acquired sAVF. Our two cases might represent a new subtype of sAVF. Based on our experiences, we recommend resection of the tumor containing the shunt for the management of sAVF.

## Introduction

Spinal lipoma is a congenital condition resulting from a disturbance in the separation of the neuroectoderm from the cutaneous ectoderm in the embryonic stage [1]. It is frequently associated with spina bifida, and produces neurological symptoms, usually in childhood by tethering of the spinal cord. Surgical treatment such as untethering of the spinal cord and debulking of the lipoma is recommended in such cases [2, 3]. Spinal arteriovenous fistula (sAVF) is a congenital or acquired shunt that forms within the spinal arterial and venous systems that can cause spinal cord symptoms because of hemorrhage or disturbance of perfusion. Co-existence of these two disease states in the spine is extremely rare, and our extensive search of the literature revealed only nine similar case reports.

We recently encountered two cases of adult-onset sAVF occurring within a spinal lipoma. Here, we describe the two cases and discuss the mechanism of and optimal treatment for this condition with reference to nine similar cases identified in review the literature [4–12].

## Case presentation

### Case 1

**Fig. 1:**
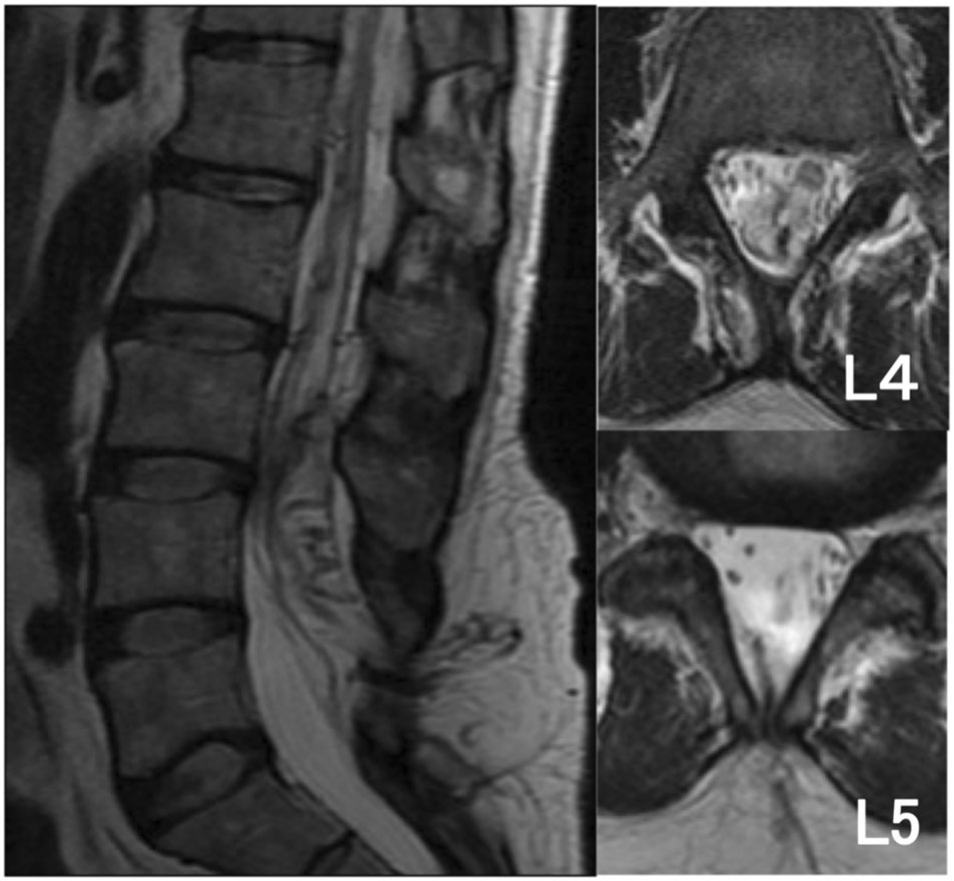
Case 1. T2-weighted MRI scan showing a spinal lipoma at the L5 level with a flow void dorsal to the spinal cord

**Fig. 2:**
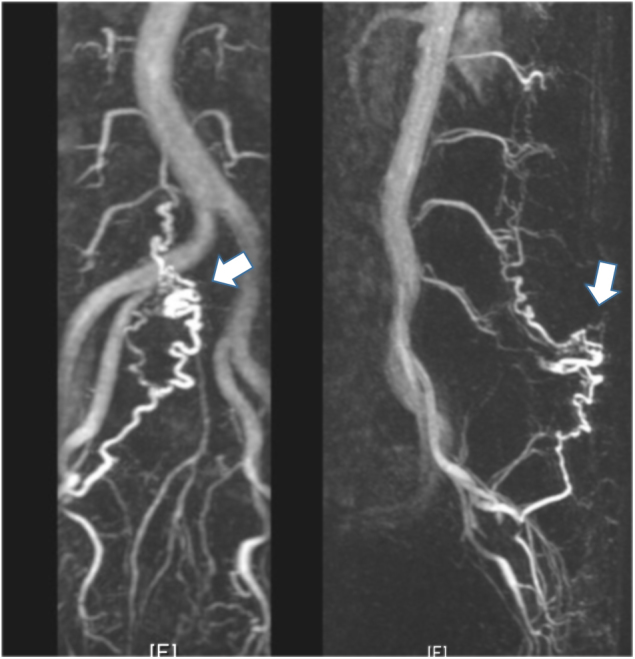
Case 1. MRA image taken with the TRICKS (Time Resolved Imaging of Contrast KineticS) sequence shows tortuous blood vessels (white arrow) at the L5 level. This area is consistent with that of the spinal lipoma shown on T2WI (see Fig. 1)

**Fig. 3:**
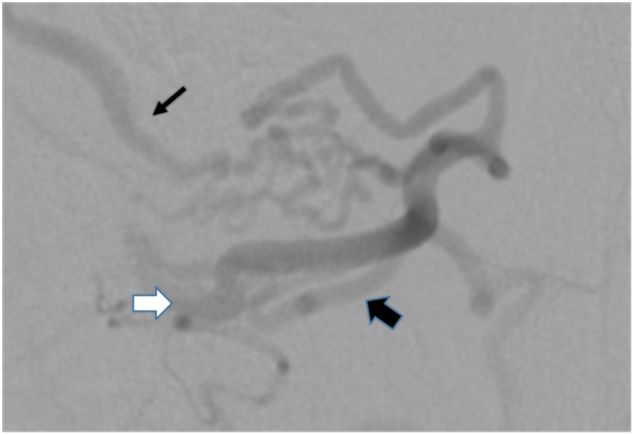
Case 1. Angiogram showing an abnormal blood vessel, with a shunt within the lipoma fed by the right sacral artery. The feeding artery (thick black arrow) can be seen entering the lipoma from the dorsal side, and passing the shunt(white arrow), and then entering the spinal canal via the juxtamedullary vein (thin black arrow)

A 51-year-old man with no significant past medical history was admitted to our hospital because of gait disturbance and dysuria. He had a 2-year history of low back pain and numbness in both legs. His symptoms worsened 1 week earlier and he suddenly developed gait disturbance associated with bilateral leg weakness. On admission, manual muscle testing revealed paresis with distal dominance. Patellar tendon and Achilles tendon reflexes were not exaggerated. He had urinary retention and his lumbar Japanese Orthopedic Association (JOA) score was only 2/29, indicating severe physical impairment (the lower the score, the more severe the impairment). MRI of the spine revealed a spinal lipoma at the L5 level and a flow void dorsal to the spinal cord at approximately the level of the conus medullaris (Fig. 1). MRA revealed tortuous blood vessels within the spinal lipoma (Fig. 2). Subsequent angiography showed an arteriovenous shunt from the right lateral sacral artery. The feeding artery entered from the dorsal aspect into the lipoma, and then passed the shun zone, and entered the spinal canal primarily via the juxtamedullary vein (Fig. 3).

Diagnosis was a sAVF within a spinal lipoma, and the patient underwent ligation of the draining vein combined with embolization. Under general anesthesia, a catheter was inserted into the lateral sacral artery and advanced to just before the shunt of the fistula. Because a provocation test with lidocaine hydrochloride (hereafter, xylocaine test) showed depression of the motor-evoked potentials (MEP) in the left anterior tibial and anal muscles, embolization using any liquid embolic agent was deemed to be high risk and, we performed only proximal artery occlusion with a coil. Angiography of the internal iliac artery after embolization revealed slight remaining density in the shunt. Therefore, ligation of the draining vein was additionally performed under microscopic view, with transection of two draining veins in the lipoma. Postoperative angiography confirmed there was no remaining shunt.

**Fig. 4:**
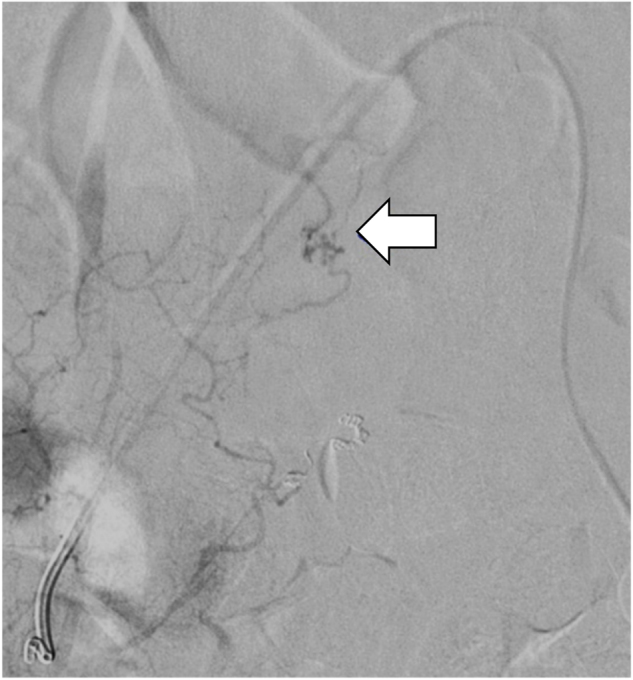
Case 1. Angiogram at 2 months after the surgery showing an arteriovenous shunt (white arrow) mediated by a collateral route from the right lateral sacral artery

Postoperatively, bilateral lower limb muscle strength was slightly improved, but there was no appreciable improvement in vesico–rectal disturbances. At 1 month after surgery, his lumbar JOA score had improved to 10/29, but angiography revealed an arteriovenous shunt via a collateral route from the right lateral sacral artery (Fig. 4). The patient experienced no aggravation of symptoms thereafter. Although his low back pain, leg numbness, and bladder dysfunction persisted, intermittent claudication, muscle weakness, and the ADL score were improved at 1 year after surgery. His lumbar JOA score was further improved to 13/29.

### Case 2

**Fig. 5:**
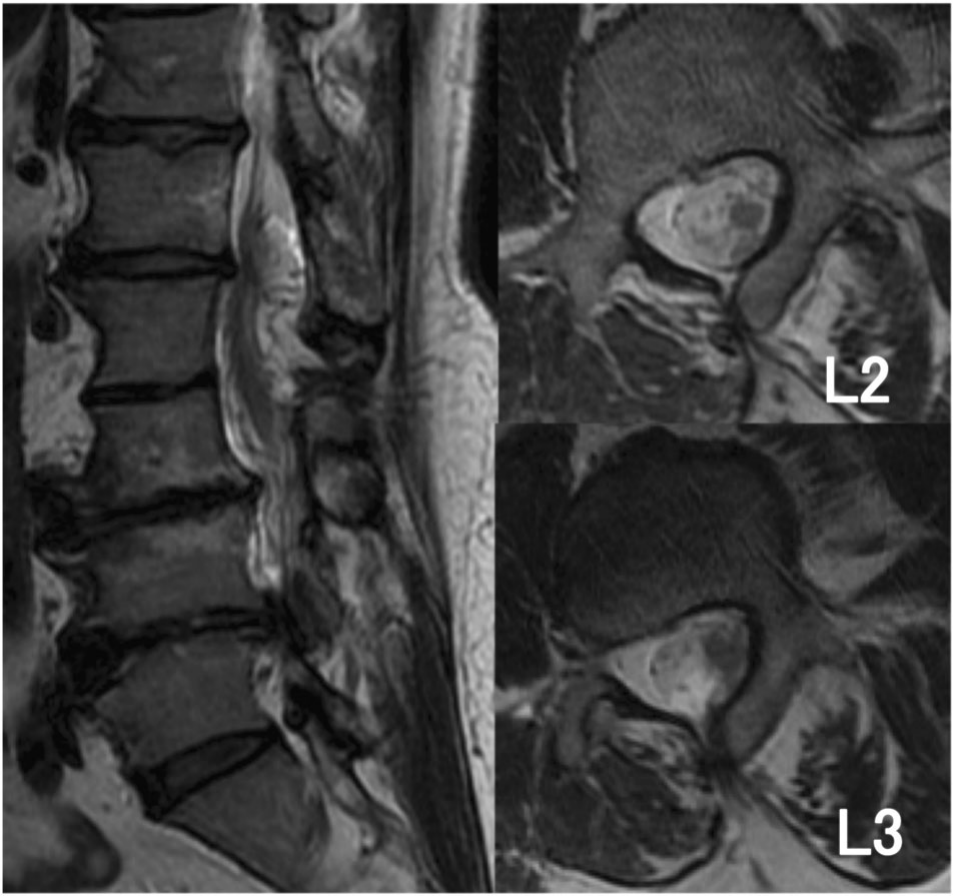
Case 2. T2-weighted MRI scans showing a spinal lipoma at the L2/3 level with a flow void dorsal to the spinal cord

**Fig. 6:**
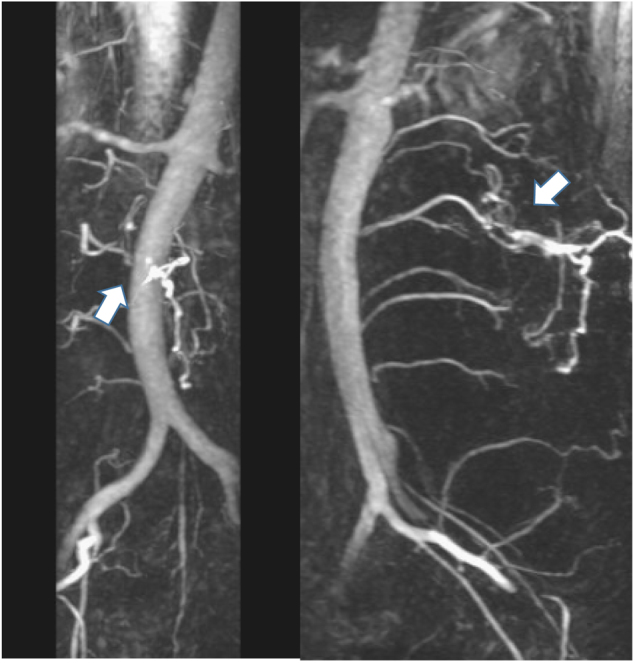
Case 2. MRA images taken with the TRICKS sequence shows tortuous blood vessels (white arrows) at the L2/3 level. This area is consistent with where this patient’s spinal lipoma was identified on T2WI (see Fig. 5)

**Fig. 7:**
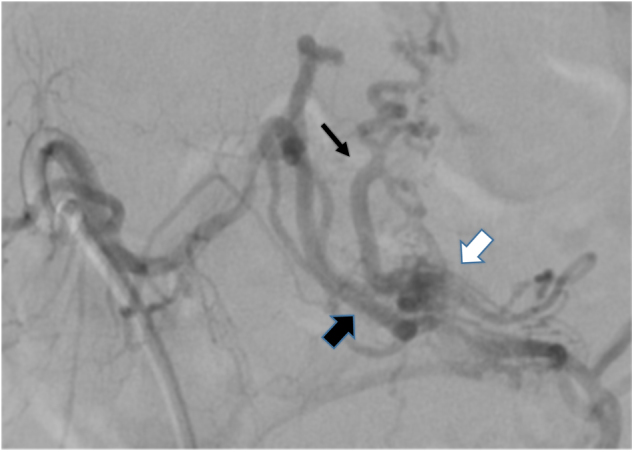
Case 2 Angiogram showing an abnormal blood vessel with a shunt within the lipoma fed by the right second lumbar artery. The feeding artery (thick black arrow) is entering the lipoma from the ventral side, passing the shunt (white arrow), and then entering the spinal canal as the draining vein (thin black arrow)

A 53-year-old man with no significant past medical history was admitted to our hospital because of pain in the lower-extremities and gait disturbance. His gait disturbance noticed by his family when he was 30 years of age. At age 51, he was found to have a spinal lipoma at a neighboring hospital and was referred to our institution. His lower extremity pain and gait disturbance worsened while he was under observation and he was admitted for further examination. Manual muscle testing revealed lower limb paresis that was more pronounced on the right side. The patellar tendon reflex was not exaggerated on either side, but the Achilles tendon reflex was hyperactive and the Babinski sign was positive on both sides. There was no evidence of bladder or bowel disturbance, and the lumbar JOA score was 10/29. MRI of the spine revealed a spinal lipoma at the L2/3 level and a flow void dorsal to the spinal cord at the level of the conus medullaris (Fig. 5). MRA revealed tortuous blood vessels within the spinal lipoma, as in Case 1 (Fig. 6). Subsequent angiography revealed an arteriovenous shunt from the right second lumbar artery. The radiculopial artery entered the spinal canal from its ventral aspect to form a arterio-venous shunt within the lipoma. A vein from the shunt region drained into the juxtamedullary vein within the spinal canal, and formed a congestion of the venous circulation (Fig. 7).

Diagnosis was sAVF arising within the spinal lipoma, and embolization and possible surgery was scheduled. First, embolization was performed for the second lumbar artery, the feeding artery for the shunt. A xylocaine test at the feeding artery showed transient depression of MEP (as in Case 1) in the right leg muscles. However, we carried out embolization therapy as we judged the risk would be low as long as embolization was confined solely within the shunt. Because of the presence of multiple feeding arteries, the minor feeder was embolized with a coil first and then N-butyl-cyanoacrylate was injected through one of the main feeders into the shunt zone to occlude it. This did not achieve complete embolization, and surgical excision was deemed necessary. The shunt-containing lipoma was resected as far as possible. An abundance of blood vessels was confirmed within the lipoma. Intraoperative angiography confirmed there was no remaining shunt after the resection.

After surgery, lower limb pain and muscle strength were improved on both sides. At 1month after surgery, lumbar JOA score was 14/29. At 1 year, angiographic examination showed no vascular abnormalities. Although the patient’s low back pain and leg numbness persisted, muscle strength and ADL score were improved. His lumbar JOA score was further improved to 17/29.

## Discussion

### Pathophysiology

Spinal lipomas have a predilection for the lumbodorsal region, and they develop because of disrupted separation of the neuroectoderm from the cutaneous ectoderm and fusion of the neuroectoderm with adipose tissue, which is of mesodermal origin [1]. These lipomas are often diagnosed at birth based on skin manifestations, such as the presence of a lumbosacral mass or a dermal sinus, and symptoms frequently develop in childhood due to progressive spinal cord tethering.

In 1982, spinal lipomas were classified by Chapman into three types, dorsal, caudal, and transitional according to the position of the tumor in relation to the spinal cord [2]. In 2001, Arai et al. proposed a classification into five types, the three conventional types (dorsal, caudal, and combined), a filar type involving the origin of the filum terminale, and lipomyelomeningocele(LMMC), in which the spinal cord and neural tissue deviates out of the spinal canal [3]. In a 2010 report of 175 cases of spinal lipomas by Nomura et al., 21.1% of cases were transitional type, 20% were LMMC type, 17.7% were dorsal type, 14.3% were caudal type, 13% were filar type [13]. In both of our cases, the spinal lipoma was dorsal type. In the nine cases of spinal lipoma with concurrent sAVF reported in the past, the dorsal type was present in five cases, the combined type in one case, the filar type in two cases, and the LMMC type in one case. Thus, the type of spinal lipoma varied, but the dorsal type was the most common.

Spinal AVF is a condition in which circulating blood in the arterial system passes directly into a draining vein that would normally perfuse the tissues by passing into the capillaries before it enters the venous system. Eventually, the elevated venous blood pressure impairs perfusion of the spinal cord, and produces symptoms associated with ischemia and bleeding. In 2000, Miyasaka et al. classified sAVFs on the basis of their vascular anatomical significance and options for treatment into three types: dural AVF, perimedullary AVF, and intramedullary arteriovenous malformation (AVM) [14]. In 2002, Rodesch et al. proposed a classification system where arteriovenous fistulas were classified as AVM and AVF, with each type subdivided further into microfistula and macrofistula according to whether or not it could be treated by intravascular surgery [15]. In the paper by Rodesch et al, reference is made to epidural AVF and paraspinal AVF (which are not included in Miyasaka et al.’s classification), and to a disease state involving concurrent vascular anomalies. It could not be clearly determined whether the condition in cases was intradural or extradural because both patients lacked a dura on the dorsal side of the fistula adjacent to the lipoma, and there were no concurrent vascular malformations. Therefore it is considered that our two cases and the previously reported nine cases represent a novel type of sAVF that does not fall under the previously published classifications. The location of the shunt essentially coincided with that of the lipoma in the cases reported in the past, so it would be reasonable to infer that the shunt was located within the lipoma, even in those cases in which there was no information on the location of the shunt. The feeding artery varied from case to case, and the blood vessel serving as the feeding artery might be identified according to the level of the spinal cord at which the lipoma was situated.

### Mechanism for AVF Development in a Spinal Lipoma

The mechanism underlying the development of an AVF within a spinal lipoma has been extensively discussed in past reports, but no consensus has been reached. Weon et al. consider this disease state to be congenital, in that the mesoderm has a bearing on disturbance of neural tube closure and angioblasts differentiate from the mesoderm during the same phase [4]. Three of the previous case reports mentioned a pathologic diagnosis for the excised tumor in all reports, it was stated that vascular lesions, such as shunts, could not be identified despite the fact that pathologic examination of the spinal lipoma confirmed thickened venous wall [5–7]. Thickening of the wall of the draining vein was apparent in our Case 1. Common to all the previous reports was a dilated venous vessel with a thickened wall, suggestive of the presence of venous hypertension. However, no evidence of congenital vascular malformation or any shunt zone has been reported as yet, so sAVF in this disease state have not as yet been confirmed to be congenital.

A close association has been reported between acquired AVF and venous hypertension [16, 17]. Animal studies have demonstrated that angiogenic factors are strongly involved in the development of dural AVF [18, 19]. The putative mechanism was suggested to include venous hypertension leading to tissue ischemia and release of angiogenic factors from the ischemic tissue into the blood, generating an acquired shunts. These experiments pertained to AVFs in the brain, and a similar mechanism may be involved in the development of spinal AVFs.

The presence of a shunt within a spinal lipoma was the most remarkable feature observed in our patients. Lipomas can liberate angiogenic factors such as fibroblast growth factor-2 and vascular endothelial growth factor, giving rise to angiogenesis within the lipoma [20]. Abundant vascular proliferation in the lipoma was confirmed intraoperatively in past reports, and it would be reasonable to assume the specific mechanism in our two cases involved, angiogenic factors liberated within the lipoma inducing angiogenesis and giving rise to an acquired AVF, rather than the usual mechanism involving venous hypertension.

### Treatment

Treatment in the nine previously reported cases [4–12], consisted of embolization alone in one case, surgery alone in three cases, and surgery with preceding embolization in five cases (Table 1). All patients identified to date had an uneventful postoperative course. However in our Case 1, in whom neither the lipoma nor the shunt zone was resected, a vascular abnormality was visualized in a collateral shunt on angiography at 1 month after surgery. Appearance of this vascular abnormality on imaging was probably attributable to re-dilatation of a latent shunt or draining vein despite the draining vein having been ligated, given that the disease state in this patient did not include a single draining vein,as is the case with a spinal dural AVF. There is a potential risk of symptomatic recurrence in this patient, so he is being followed up closely. In both our cases a xylocaine test at the feeding artery showed depression of MEP at the time of embolization, suggesting a complex vasculature within the lipoma. Therefore, it is difficult at present to accomplish radical embolization including the shunt zone upon delineation of the entire profile of the vascular lesion. Accumulation of cases in the future and more practical experience is needed.

**Table 1:** Summary of previously reported cases [6–9, 15–19]Please check table 1 is ok?OK.Thank you for improving it.

Pt.	Authors	Age	Lipoma type	Tumor level	AVF type	Feeder*	Therapy		Results
		Gender					Embolization	Surgical operation	
1	M Djindjan	53	Filum terminale	S2-3	Dural AVF	M,Lt LSA	○	○	Improved
	-1989	Male							
2	M Konig	50	Lipomyelocele	L5	Dural AVF in lipoma	Lt L3 LA		○	Unknown
	-1999	Male							
3	JH Lee	44	Dorsal type	Th11-12	Intradural AVM in Lipoma	Rt T12,Lt T10 ICA	○	○	Complete recovery
	-2000	Male				Lt L1 LA			
4	C Weon	30	Dorsal type	L4/5	Intramedullary AVM in Lipoma(L3/4)	Lt L3.L4 LA	○	○	Improved
	-2005	Male				Rt L3 LA			
5	C Cheung	42	Filum terminale	S1-2	intradural AVM In Lipoma	ASA	○	○	Improved
	-2005	Male							
6	K Rajeav	44 Female	Dorsal type	L1-2	Dural AVF	Lt L1 LA		○	Improved
	-2005				L1/2 Foramen				
7	C Erdogan	40	Dorsal type	L3	Dural AVF in lipoma	Rt L2,	○	○	Complete recovery
	-2007	Male				Lt L3 LA			
8	M Sato	72	Dorsal type	L3	Dural AVF	Rt L2 LA	○		Improved
	-2013	Male			L3/4				
9	S.B.Mavani	29	Traditional type	L5/S1	Dural AVF	Lt L4 LA		○	Improved
	-2014	Male			L4/5				
10	case (1)	51	Dorsal type	L5	AVF in llipoma	Rt LSA	○	○	Improved
	-2016	Male							
11	case (2)	53	Dorsal type	L2-3	AVF in lipoma	Rt L2 LA	○	○	Improved
	-2016	Male							

All were adult-onset cases, and the feeding artery for the AVF varied according to the level of the spinal lipoma. Although the types of AVF differed, the presence of a shunt within the spinal lipoma was a common feature

*LSA* lumbosacral artery, *LA* lumbar artery, *ICA* intercostal artery, *ASA* anterior spinal artery

The two diseases entities produce very similar symptoms, so it is difficult to determine which of them causes the symptoms. Of the 11 cases reported until date (including our two cases), 10 presented with slowly progressive lower limb paralysis in adulthood; therefore, venous hypertension caused by sAVF is considered to be an essential feature of this coexistent disease state. Since possible development of symptoms arising from tethering and a mass effect of the lipoma in the future cannot be ruled out, radical resection of the lipoma together with the shunt zone is recommended. Concomitant embolization helps to decrease intraoperative blood loss and should be considered when preparing for surgical intervention, so a detailed preoperative angiographic exploration of the vascular architectures possible.

## Conclusion

We encountered two cases of a sAVF developing within a spinal lipoma. The existence of a spinal lipoma is a potential cause of an acquired sAVF and such cases might represent a new subtype of sAVF. Resection of a lipoma containing the shunt and concomitant embolization are recommended. This coexisting disease state is still not widely recognized, and it is very likely that concurrent sAVF has been overlooked in many cases of spinal lipoma. For precise diagnosis of adult-onset spinal lipoma, the possibility of this disease state should be borne in mind and the patient should be checked by MRA for tortuous blood vessels around the spinal cord. Angiographic examination should be undertaken immediately if any vascular abnormalities are identified.

## References

[1] Naidich TP, McLone DG, Mutluer S (1983). A new understanding of dorsal dysraphism with lipoma (lipomyeloschisis): radiologic evaluation and surgical correction. AJR Am J Roentgenol.

[2] Chapman PH (1982). Congenital intraspinal lipomas: anatomic considerations and surgical treatment. Child’s Brain.

[3] Arai H, Sato K, Okuda O, Miyajima M, Hishii M, Nakanishi H (2001). Surgical experience of 120 patients with lumbosacral lipomas. Acta Neurochir (Wien).

[4] Weon YC, Chung JI, Roh HG, Eoh W, Byun HS (2005). Combined spinal intramedullary arteriovenous malformation and lipomyelomeningocele. Neuroradiology.

[5] Cheung AC, Kalkanis SN, Ogilvy CS (2005). Paraplegia after tethered cord surgery: an uncommon combined anomaly of spinal arteriovenous fistula and sacral lipoma—Case report. Neurosurgery.

[6] Djindjian M, Ayache P, Brugieres P, Poirier J (1989). Sacral lipoma of the filum terminale with dural arteriovenous fistula. Case report. J Neurosurg.

[7] Lee JH, Chung CK, Choe G, Chi JG, Chang KH, Kim HJ (2000). Combined anomaly of intramedullary arteriovenous malformation and lipomyelomeningocele. AJNR Am J Neuroradiol.

[8] Konig M, Hentsch A, Schmieder K, Harders A, Heuser L (1999). Extraspinal dural arteriovenous fistula in a patient with lipomyelodysplasia: value of MRI and MRA. Neuroradiology.

[9] Rajeev K, Panikar D (2005). Dural arteriovenous fistula coexisting with a lumbar lipomeningocele. J Neurosurg Spine.

[10] Erdogan C, Hakyemez B, Arat A, Kocaeli H, Bakar A, Parlak M (2007). Spinal dural arteriovenous fistula in a case with lipomyelodysplasia. Br J Radiol.

[11] Sato M, Takigawa T, Shiigai M, Tamura G, Masumoto T, Nakai Y (2013). Spinal dural arteriovenous fistula with lipomyelodysplasia. Neurol Med Chir (Tokyo).

[12] Mavani SB, Nadkarni TD (2014). Tethered cord due to caudal lipomeningocele associated with a lumbar dural arteriovenous fistula. J Neurosurg Spine.

[13] Nomura S, Oi S, Arai H, Nagasaki M, Shirane R, Inagaki T (2010). Spinal Bifida-Analasys of Multicenter Study of COE. Nervous System in Children.

[14] Miyasaka K, Asano T, Ushikoshi S, Hida K, Koyanagi I (2000). Vascular anatomy of the spinal cord and classification of spinal arteriovenous malformations. Interventional Neuroradiology.

[15] Rodesch G, Lasjaunias P (2003). Spinal cord arteriovenous shunts: from imaging to management. Eur J Radiol.

[16] Herman JM, Spetzler RF, Bederson JB, Kurbat JM, Zabramski JM (1995). Genesis of a dural arteriovenous malformation in a rat model. J Neurosurg.

[17] Terada T, Higashida RT, Halbach VV, Dowd CF, Tsuura M, Komai N (1994). Development of acquired arteriovenous fistulas in rats due to venous hypertension. J Neurosurg.

[18] Li Q, Zhang Q, Huang QH, Fang YB, Zhang ZL, Xu Y (2014). A pivotal role of the vascular endothelial growth factor signaling pathway in the formation of venous hypertension-induced dural arteriovenous fistulas. Mol Med Rep..

[19] Uranishi R, Nakase H, Sakaki T (1999). Expression of angiogenic growth factors in dural arteriovenous fistula. J Neurosurg.

[20] Lucarelli E, Sangiorgi L, Benassi S, Donati D, Gobbi GA, Picci P (1999). Angiogenesis in lipoma: an experimental study in the chick embryo chorioallantoic membrane. Int J Mol Med.

